# Burnout Among Labor and Birth Providers in Northern Tanzania: A Mixed‐Methods Study

**DOI:** 10.1002/puh2.70014

**Published:** 2024-12-05

**Authors:** Virginie Marchand, Melissa H. Watt, Linda M. Minja, Mariam L. Barabara, Olivia R. Hanson, Janeth Mlay, Maya J. Stephens, Blandina T. Mmbaga, Susanna R. Cohen

**Affiliations:** ^1^ Department of Obstetrics and Gynecology NYU Langone Health New York New York USA; ^2^ Department of Population Health Sciences University of Utah Salt Lake City Utah USA; ^3^ Kilimanjaro Clinical Research Institute Moshi Tanzania; ^4^ Kilimanjaro Christian Medical University College Moshi Tanzania; ^5^ Department of Obstetrics and Gynecology University of Utah Salt Lake City Utah USA

**Keywords:** burnout, health workforce, labor and delivery providers, maternal health services, professional, Tanzania

## Abstract

**Background:**

Burnout, characterized by emotional exhaustion, depersonalization, and a diminished sense of accomplishment, is a serious problem among healthcare workers. Burnout negatively impacts provider well‐being, patient outcomes, and healthcare systems globally and is especially worrisome in settings with shortages of healthcare workers and resources.

**Methods:**

This study explores the experience of burnout among labor and delivery (L&D) providers in Tanzania, using three data sources. A structured assessment of burnout was collected at four timepoints from a sample of 60 L&D providers in 6 clinics. The same providers participated in an interactive group activity from which we drew observational prevalence data. Finally, we conducted in‐depth interviews (IDIs) with 15 providers to further explore their experience of burnout.

**Results:**

Prior to any introduction to the concept, 18% of respondents met criteria for burnout. Immediately after a discussion and activity on burnout, 62% of providers met criteria. One and 3 months later, 29% and 33% of providers met criteria, respectively. In IDIs, participants saw the lack of understanding of burnout as the cause for low baseline rates and attributed the subsequent decrease in burnout to newly acquired coping strategies. The activity helped them realize they were not alone in their experience of burnout. High patient load, low staffing, limited resources, and low pay emerged as contributing factors.

**Conclusion:**

A lack of exposure to the concept of burnout leads to providers being unaware of the issue as a collective burden. Therefore, burnout remains rarely discussed and not addressed, thus continuing to impact provider and patient health.

## Graphical abstract

Burnout is prevalent among labor and delivery providers in Tanzania; however, there is a lack of exposure to the concept. Through group discussions and activities, providers can recognize the issue as a collective burden and take the first steps toward addressing the root causes of burnout.

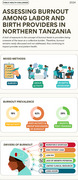

## 1 Introduction

The COVID‐19 pandemic focused international attention on healthcare provider burnout [1, 2], but it has been a chronic problem in the healthcare setting due to high levels of stress and emotional intensity associated with the job [3]. Provider burnout is characterized by three components: (1) emotional exhaustion, defined as the feeling of being “used up” and unavailable emotionally for patients at the end of the workday; (2) depersonalization, or increased callousness toward patients; and (3) a sense of diminished personal accomplishment, including feelings of ineffectiveness and lack of value to patient care [3].

Rates of burnout are higher among healthcare trainees and professionals compared to other professions [3, 4]. Burnout negatively impacts provider health and well‐being, contributing to an increased risk of depression [3, 5], alcohol misuse [6], and suicidal ideation [7]. Provider burnout also has a significant impact on patient care and the healthcare system as a whole [3], with data showing a greater rate of medical errors [8], decreased productivity [9], increased desire to quit the profession [10], and diminished rapport with patients [11].

Burnout in low‐ and middle‐income countries (LMICs), especially in Sub‐Saharan Africa, has been underexplored [12, 13, 14, 15, 16], and specific measures to assess provider burnout have not been validated. A systematic review of burnout among healthcare providers in Africa found that burnout was associated with a heavy workload, difficult work conditions, inadequate personnel, and low work satisfaction [12]. A survey of burnout among East African nurses found a high rate of emotional exhaustion and depersonalization [17]. In LMICs, the shortage of healthcare workers puts additional pressure on health systems [18]; in Sub‐Saharan Africa, nearly one‐fourth of the global disease burden is met by only 3% of the global work force [19].

Labor and delivery (L&D) wards are particularly high‐stress environments, and research suggests that maternal health staff may be at higher risk of burnout compared to colleagues in other specialties [20]. Given the impact of provider burnout on patient care, this could have important implications for maternal and child health outcomes.

Although healthcare provider burnout has been part of the national conversation in the United States in the previous 3 years, it has not received the same level of attention in LMICs. The goal of this study was to explore burnout in a population of L&D providers in Tanzania and its impact on their work. We draw on structured assessments of burnout at four timepoints, observations during an interactive group activity, and semi‐structured interviews. The findings shed light on the experience of burnout in Tanzania and can help provide guidance on measurement and intervention.

## 2 Methods

### 2.1 Overview

This mixed‐methods analysis was nested in a larger intervention study (NCT05271903), where our team engaged with providers, patients, and stakeholders to develop, deliver, and evaluate the MAMA training focused on respectful maternity care (RMC) during L&D for women living with HIV in Tanzania [21, 22]. The 2.5‐day training included case‐based learning sessions, simulations, and interactive activities on teamwork and communication, clinical empathy, stigma and bias, and RMC. Further intervention content has been described elsewhere [21].

In speaking with providers while developing the intervention, we uncovered burnout as a possible contributor to suboptimal delivery of RMC and an actionable area for intervention. Therefore, we decided to incorporate assessments of, and content material on, burnout throughout the training.

### 2.2 Study Setting and Population

Study participants were 60 L&D providers from 6 primary care hospitals in the Moshi (urban) and Rombo (rural) districts of the Kilimanjaro region of northern Tanzania. Doctors, clinical officers, and nurse‐midwives working in L&D departments were eligible to participate. The sample size of 60 was based on consultation with the Tanzania Ministry of Health to ensure adequate intervention coverage while not disrupting clinical flow. Leadership at each of the 6 study sites was asked to identify 10 interprofessional team members to attend. The final sample represented 62% of all eligible providers across the study clinics.

When participating providers reported to the training, they were given an informed consent form to review and had the opportunity to ask questions prior to signing. Upon providing informed consent, participants were assigned a study identification number. The participant flow and follow‐up rate are visualized in Supporting Information 1.

We explored burnout through three data sources in this mixed‐methods study: observational data from an interactive activity on burnout during the MAMA training, structured survey assessments of burnout, and in‐depth interviews.

### 2.3 Study Variables, Instruments, and Data Collection

#### 2.3.1 Interactive Session

The MAMA training was delivered to providers in November 2022. The curriculum included a 45‐min session focused specifically on burnout, followed by three sessions on clinical empathy, bias and stigma, and mindfulness and coping strategies. We first defined burnout for participants as a combination of emotional exhaustion, depersonalization, and diminution of personal accomplishment, explaining this could present as feelings of emptiness, fatigue, negative or distant attitudes toward others, feelings of failure, or low self‐esteem. We prompted providers to share their thoughts on factors contributing to burnout in their clinical settings and to engage in a discussion based on their experience. Then, we invited them to line up along a scale from “not burned out at all” to “completely burned out” to create a visual representation of the prevalence of burnout among participants. The purpose of the activity was to develop a shared understanding of burnout in their clinical settings and to lay the foundation for authentic conversations.

After the activity, we discussed personal strategies to cope with stress in high‐pressure work environments, including mindfulness and clinical empathy skills. These included deep breathing, body scans, positive affirmations, holding a powerful pose, daily intentions, stretching, writing down accomplishments, focusing on sensations during tasks, and taking a moment to pause and reflect on next steps when possible. Participants had the chance to practice each one together and were encouraged to choose one or two to practice at work.

The topics of burnout, mindfulness, and empathy were integrated throughout the rest of the MAMA training, which was designed to support providers’ emotional well‐being and help them connect with themselves, their colleagues, and patients.

#### 2.3.2 Survey Assessment

We assessed provider burnout using a two‐item measure [23] adapted from the Maslach Burnout Inventory [24], which was translated, back translated, and piloted with team members and a small group of providers to ensure accurate translation. After giving a written definition of burnout (Box 1), translated as “msongomoto” in Swahili, the first question asks respondents to select from a 5‐point Likert scale: “(1) I enjoy my work. I have no symptoms of burnout”; “(2) Occasionally I am under stress, and I don't always have as much energy as I once did, but I don't feel burned out”; “(3) I am definitely burning out and have one or more symptoms of burnout, such as physical and emotional exhaustion”; “(4) the symptoms of burnout that I'm experiencing won't go away. I think about frustrations at work a lot”; and “(5) I feel completely burned out and often wonder if I can go on. I am at the point where I may need some changes or may need to seek some sort of help.”

The second question asked participants how often they feel they have become more callous toward patients since taking this job because of burnout. The response options were on a 7‐point Likert scale from “Never” to “Every Day.”

We used this measure to assess burnout at four timepoints: immediately before the delivery of the MAMA training, immediately after the burnout session and interactive activity during the training, then again 1‐ and 3‐month post‐training. Using >2 as the cutoff for burnout for the first question (on a 0–4 scale) and >4 for the second question (on a 0–6 scale), we considered providers burned out if they met criteria for burnout in either question and classified providers categorically as positive or negative for burnout for our analysis [23, 25].

**Box 1: Definition of burnout given to participants as part of the measure**

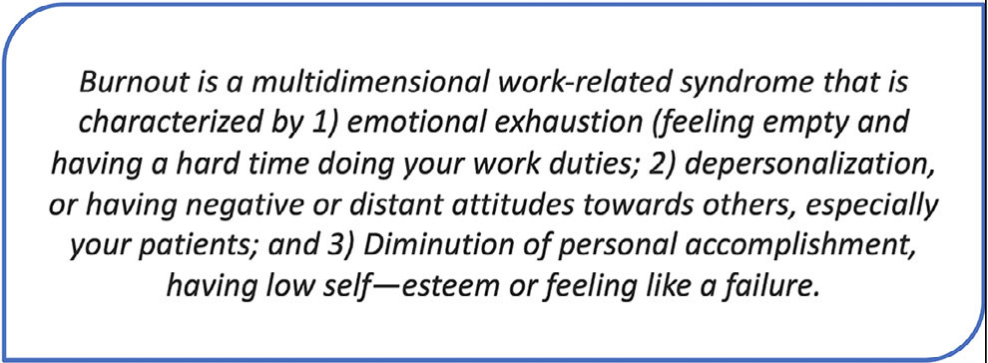

#### 2.3.3 In‐Depth Interviews

To further understand the experience of burnout in their clinical context, we invited 15 providers (2–3 from each facility) to further discuss burnout through in‐depth interviews 3 to 4 months post‐training. We selected participants who had actively participated during the MAMA training and were viewed by the team as “good informants” [26], with consideration for representation of gender, age, and profession. All 15 providers invited to participate in interviews agreed. Interviews were conducted in Swahili using a semi‐structured interview guide by 4 research team members familiar with the local context and with experience in qualitative data collection. The sub‐section of the interview guide focused on burnout is available in Supporting Information 2. During the interviews, we asked providers if they felt that burnout was common among their colleagues and how it manifests. We asked if burnout was discussed among coworkers, both before and after the training, and if so, how it is described. We also explored the impact the training had on their view of burnout in themselves and among teammates. Finally, we applied the method of member checking by presenting interviewees with our preliminary survey data on reported burnout and asking for their interpretation of the trends between time‐points based on their experience.

### 2.4 Data Analysis

Survey data were entered into REDCap software and exported to R for data analysis. We first used summary statistics to describe participant characteristics. We then determined the proportion of patients meeting criteria for burnout at each timepoint and used a Pearson's Chi Squared test to assess changes overall and from mid‐training to 1‐month post‐training. Interviews were transcribed and translated, then exported into NVivo for applied thematic analysis [27]. The data were coded to identify emerging themes across five domains: prevalence of burnout, drivers of burnout, manifestations of burnout, explanations of low baseline scores, and explanations for burnout changes over time. The research team then grouped and synthesized concepts using axial coding [27, 28]. Analysis was iterative throughout the data collection process to assess for thematic saturation [29], which was determined when no new themes or information emerged and themes were felt to adequately describe the domain of interest.

## 3 Results

### 3.1 Description of Study Participants

A total of 60 providers completed the MAMA training, which included the interactive activity on burnout. Surveys were completed by 66 participants pre‐training, 66 immediately post‐training, 55 one‐month post‐training, and 59 three‐month post‐training. Fifteen participants participated in the interviews. Table 1 describes baseline characteristics of our sample of participating providers.

**TABLE 1:** Description of the sample of labor and delivery providers participating in the MAMA intervention.

	MAMA Training participants (*n* = 60)	Interview participants (*n* = 15)
	*n* (%)	*n* (%)
**Gender**		
Female	29 (48)	7 (47)
Male	31 (52)	8 (53)
**Clinical training**		
Nurse midwife	40 (66.7)	9 (60)
Clinical officer	4 (6.7)	1 (7)
Medical doctor	16 (26.7)	5 (33)
**Number of trainings on labor and delivery for women living with HIV in the past year**		
None	46 (77)	11 (73)
1 or more	14 (23)	4 (27)
	Median (*Q*1, *Q*3)	Median (*Q*1, *Q*3)
**Age**	32 (27, 37)	32 (29, 39)
**Years since clinical training**	8 (4, 12)	6 (8, 10)
**Years providing care in labor and delivery**	5 (2, 8)	5 (4, 7)
**Years working at current clinic**	5 (1, 9)	5 (1, 9)

### 3.2 Baseline Assessment of Burnout

In the baseline surveys prior to the training, only 11 providers (18%) met the criteria for burnout. This came to the attention of the lead trainers, as the pre‐training development phase had revealed high stress, workload, and limited personnel as issues, all of which in the literature are linked to high burnout. This observation triggered the training team to expand the training's focus on burnout, to take the time to discuss the definition of burnout, to engage in a discussion of the causes and manifestations of burnout, and to lead the interactive activity described below.

### 3.3 Training Session and Burnout Activity

**FIGURE 1:**
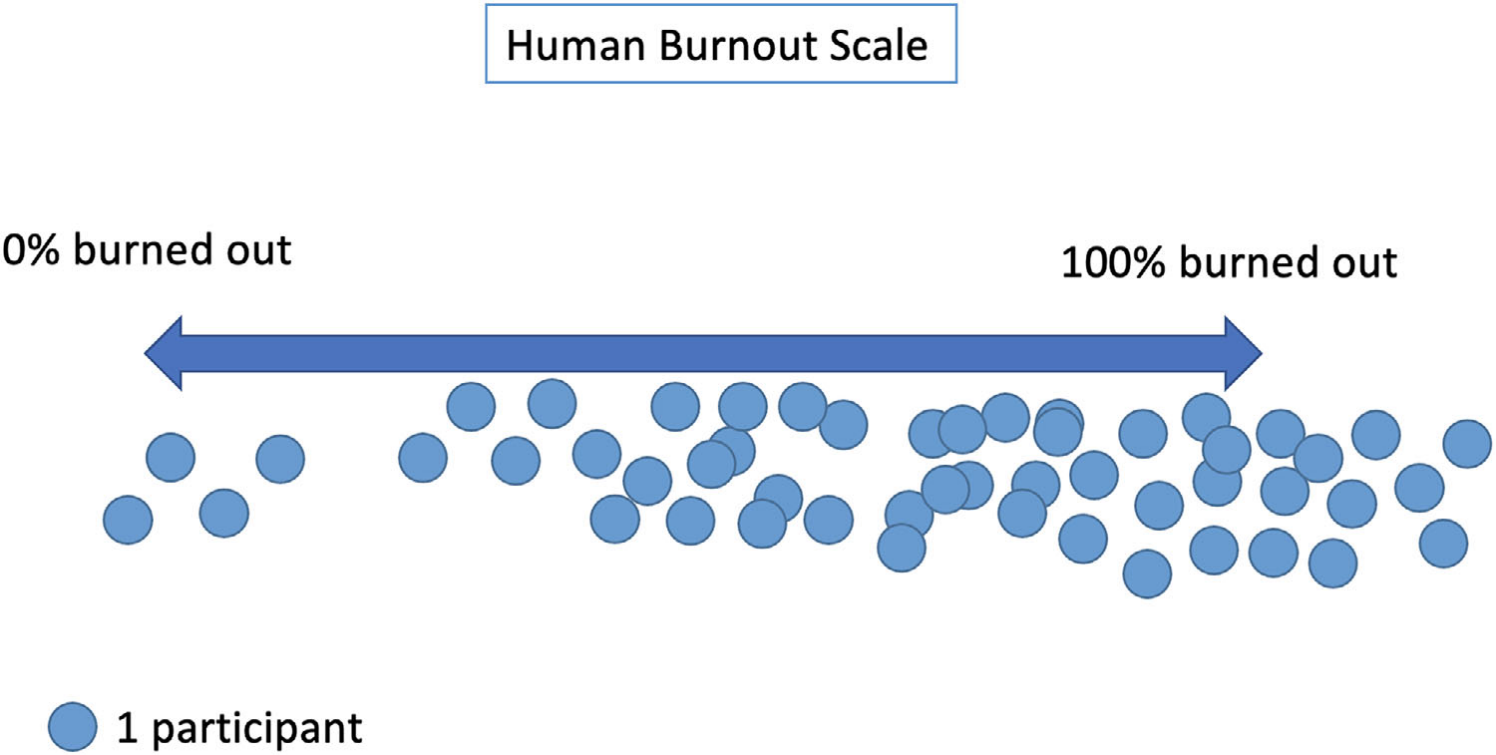
Graphic representation of participants’ placement along the human scale from “not burned out at all” to the left to “completely burned out” to the right.

After the discussion, providers were asked to physically place themselves along a continuum, from “not at all burned out” to “completely burned out.” The human scale revealed that a majority of participants felt burnt out, standing close together toward the right side of the scale (Figure 1).

### 3.4 Assessment of Burnout Over Time

**FIGURE 2:**
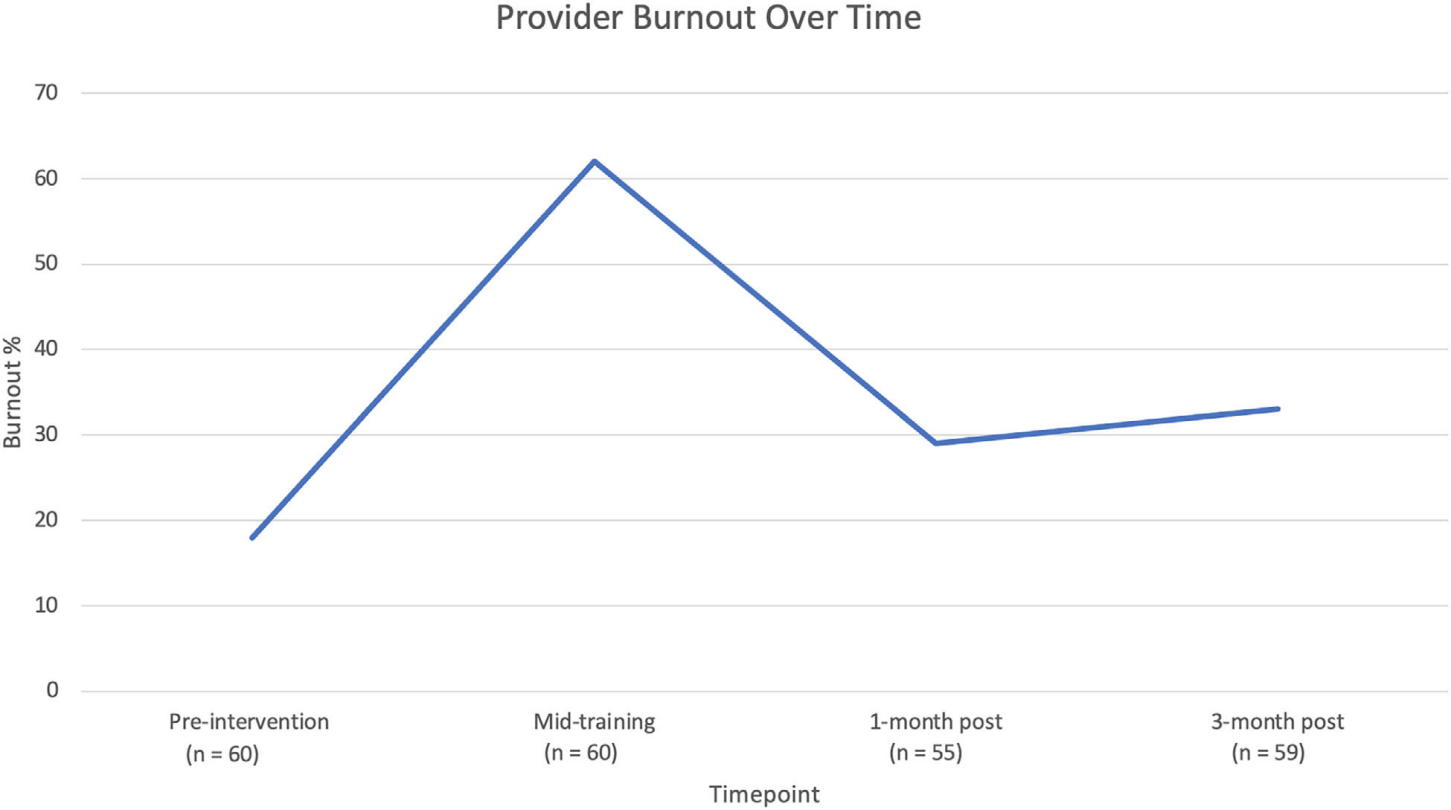
Change in proportion of providers meeting burnout criteria over time.

Survey data showed that the percentage of providers meeting criteria for burnout significantly varied across the study timepoints (*p* < 0.001), as seen in Figure 2. Immediately after the discussion of burnout and interactive activity, burnout was reassessed, and prevalence rose from 11 (18%) at baseline to 37 (62%), a number closer to what was seen in the human scale created during the activity. One month post‐training, burnout decreased significantly from the mid‐training exercise (62% vs. 29%, *p* < 0.001), with the impact sustained (33%) at 3‐month post‐training (Figure 2). A limited, de‐identified dataset is available in the repository of the Inter‐University Consortium for Political and Social Research (ICPSR‐194883).

### 3.5 In‐Depth Interview Findings

The interviews with 15 L&D providers offered additional insight into the experience of burnout in this population and an explanation of the change in burnout over time. The majority of respondents (14/15) believed that burnout was widespread in their workplace. Table 2 describes the themes that emerged relating to prevalence, drivers, and manifestations of burnout, and explanations for low baseline burnout scores and for changes in reported burnout over time.

**TABLE 2:** In‐depth interview emerging themes.

Themes	Representative quote
**Prevalence of burnout**	
High levels of burnout	“Burnout is high from the patients themselves, from the working environment, from the leadership.” (Nurse midwife, age 35)
**Drivers of burnout**	
Workforce shortages	“Shortage contributes. […] The work that should be done by three people is being done by one person.” (Nurse midwife, 40)
High patient load	“What causes [burnout] is the environment you are in and the number of patients you serve. […] Loosing hope among health providers can be caused by having so many duties. […] We have been serving many people, you are just one person, you work more than your ability.” (Nurse midwife, 35)
Long working hours	“Burnout happens if you are working so much without refreshers, or you get little rest.” (Clinical officer, 30)
Low pay	“Our level of pay is not satisfactory, and it causes burnout. You have a family, you have the relatives, you have the bills to pay, and you don't have money and you are supposed to work. I think those are the things that stress us a lot.” (Nurse midwife, 35)
Lack of growth opportunities	“Our public servants sometimes they don't get a raise in their positions–this is a very difficult thing. You find that someone is in the same position for 5 years without a raise in the position or in the salary. Therefore, things like this are outside the power of the center, but in one way (indirectly) it is affecting the effectiveness of work.” (Doctor, 52)
Home stress	“There are challenges like the increase in life cost. Everything is expensive and people have debts […] What do you expect, won't that person get burnout? […] Most women are the pillars of their home. She is carrying many responsibilities: home needs, food, children need school fees, mother is sick in the village… so the woman has many things. Patients are not the only reason that makes her feel tired.” (Nurse midwife, 28)
Blame for mistakes	“When death occurs, that's when it becomes more challenging. […] There will be a person who will ask you questions as if you did it intentionally. […] If you find yourself being pointed fingers at it discourages you and you end up coming to work just because you are obliged to.” (Doctor, 27)
**Manifestations of burnout**	
Work efficiency and quality	“You do not enjoy the work you do, you just do it. You push yourself because you must, and if it was your decision, you would leave it. Once burnout reaches to that point, a person will just go to work and wait for working hours to end. […] So the quality of service will be low. The mother is affected.” (Nurse midwife, 35)
Suboptimal respectful maternity care	“She cannot serve the mother respectively because she is burned out. So when the mother talks to her, she think the mother is disturbing her.” (Nurse midwife, 40)
Impatience with colleagues and patients	“A person might call the nurse, and the nurse answers ‘what do you want, you call too much. […] She has been working continuously, she did not get time to rest, so she considers that these patients are the one making her tired. […] Later she answers her fellow staff rudely. You will hear ‘leave me alone, I am tired with this work.’ […] You might find yourselves quarreling because you are not in a good mood.” (Nurse midwife, 40)
Anger	“It reaches a point where you lose hope, you lose energy, and even when you go back home, you feel angry when someone talks to you.” (Nurse midwife, 40)
Loss of motivation	“You will find a person lacking work motivation, the motivation becomes very low that you will find someone coming to work just because they are obliged to it and not because they are happy to come” (Doctor, 27)
Unnecessary referrals	“You might even notice that they referred the patient because they did not want to do the work” (Doctor, 27)
**Explanation of low baseline scores for burnout**	
Lack of understanding	“The burnout, people had it and it is not that they acquired it after the activity, but they never knew that it was burnout and therefore, when they found out the meaning of burnout, they admitted that they had it–that is what caused many people to admit having burnout in the end.” (Doctor, 52)
Fear of being alone	“Others are afraid to say because he might have been seen as the only one with burnout, so he decided to keep quiet.” (Nurse midwife, 40)
Fear of admitting to low‐quality work	“If someone opens up to everyone that they have burnout that means they are telling you that “my service provision is of low quality.” (Doctor, 52)
Fear of being reported	“We were worried that in this work, you want us to say this and then you report us somewhere that is why we were afraid.” (Nurse midwife, 32)
**Explanation of the change in burnout over time**	
Recognition of burnout	“A person might just have felt that he had no mood for the work […] without knowing that is burnout. After the training, people came to know that what we were feeling was burnout itself. […] So I realized that I truly have burnout.” (Nurse midwife, 35)
Realizing they are not alone	“I felt shocked because […] many people are very burned out since we are working under pressure, so I felt that I was not alone.” (Nurse midwife, 35)
Normalizing burnout	“I became aware that burnout at work is normal.” (Nurse midwife, 28)
Discussion of burnout among colleagues	“Almost every day we talk about burnout. […] When we left the training, others were wondering, ‘what is burnout?’ so, we explained it to them, and they started saying ‘aah I always get it also.’” (Nurse midwife, 40)
Impact of training	“It is not that [burnout] has ended, it has reduced because if you use those techniques of self‐releasing from stress, you become a little better, you continue with your work again. But without getting these trainings about what to do when you have stress, others could have surely quite the job.” (Nurse midwife, 40)

#### 3.5.1 Drivers of Burnout

Respondents identified workforce shortages and high patient load as the most common drivers, along with low pay, long working hours, limited resources, and a lack of opportunities for professional growth. Participants spoke about clinical factors leading to an inability to provide quality patient care. Several participants also discussed home stress and pressure from leadership as contributors to burnout.

#### 3.5.2 Manifestations of Burnout

Most respondents highlighted the impact of burnout on work efficiency and quality. Other manifestations of burnout that emerged included suboptimal RMC, impatience both with colleagues and patients, loss of motivation, and anger. One interviewee pointed to an increased number of unnecessary referrals.

#### 3.5.3 Explanation for Low Baseline Scores

When asked why baseline (pre‐activity) burnout scores might have been low despite experiences of burnout among providers, participants all responded that this was due to a lack of understanding of the term “burnout” and unfamiliarity with the concept. A few interviewees also suggested respondents may have felt reluctant to share initially due to a fear of being the only one experiencing burnout, feeling they would be admitting to low‐quality work, or a fear of being reported to leadership by the study team.

#### 3.5.4 Explanation of Changes in Reported Burnout Over Time

A majority of interviewees said the activity enabled providers to recognize burnout as an issue, to realize they were not alone experiencing the feelings of burnout, and to normalize burnout as a common issue among their colleagues, leading to an increase in burnout levels reported immediately after the training activity. The training also generated further discussion around burnout upon returning to work, and all interviewees emphasized how useful the coping strategies discussed during the training had been in helping them manage their stress. They all attributed the decrease in burnout prevalence seen at 1‐ and 3‐month post‐training to the impact of the training and use of the skills they acquired there.

## 4 Discussion

Burnout among healthcare workers in Sub‐Saharan Africa remains underexplored and poorly understood. However, its impact on provider well‐being, patient outcomes, and healthcare systems is undeniable. In this article, we present the findings from our study of provider burnout among L&D providers in Tanzania explored through survey assessments, a team activity, and in‐depth interviews. Results speak to the need for better assessment of burnout for monitoring and evaluation and for identifying intervention approaches to address this issue.

### 4.1 Measuring Burnout

To understand the burden of burnout, we must first be able to identify and measure it using validated tools. The survey we used was an established two‐item measure of burnout [24]. At baseline, before the training session on burnout, most providers scored low on the burnout scale, with 18% meeting criteria for burnout, which was not consistent with our clinical observations and discussions with providers. During the training, we realized that most participants had not previously heard of the concept of burnout, consistent with previous reports of the under‐identification of burnout in LMIC [12]. This was confirmed during our in‐depth interviews, in which providers all expressed that prior to the training, burnout was not a concept they were familiar with, despite experiencing the symptoms of burnout. The use of the activity to define and discuss burnout gave the providers a “name” for feelings they were already experiencing.

Despite their recent introduction to the concept of burnout, providers were already aware of the causes and consequences of the exhaustion they were experiencing. Throughout in‐depth interviews, they shared that workforce shortages [18] and high workloads [12] place an immense burden on healthcare providers, leading to decreased efficiency and productivity [9], diminished RMC and rapport with patients [11], and negative impacts on work quality and the healthcare system [3], all of which have been reported in previous literature.

Following the discussion and activity, participants’ scores on the repeated burnout scale were significantly higher, with 62% meeting criteria for burnout. When discussing this change during the interviews, providers said this rise in reported burnout represented a new understanding of the term and a recognition of pre‐existing symptoms as burnout. This experience suggests that brief measures of burnout may not be valid without first explaining and discussing the concept. In contrast, other studies using longer and more comprehensive assessments of burnout have identified higher rates of burnout, even in populations that may be unfamiliar with burnout as a concept [12].

### 4.2 Addressing Burnout

Providers identified workforce shortages, high patient load, low pay, long hours, and limited resources as causes of burnout, which are recurring themes in previous literature on burnout in Sub‐Saharan Africa and clearly not modifiable through provider training [12, 18]. However, one critical beginning step in addressing burnout is enabling providers to recognize it as an issue. The interactive activity showed that a majority of providers were experiencing symptoms of burnout, and this helped providers see that they were not alone in their feelings of exhaustion and depersonalization due to their job, but that this was an experience shared by colleagues. This was an important step in recognizing the issue as collective and systemic. This process demonstrates the importance of identifying burnout and encouraging discussion among providers for it to be recognized and addressed. These findings are consistent with previous research, which has shown that sharing personal experiences with peers reduces professional isolation [30] and that interventions promoting collegiality, shared experience, and community among healthcare providers can improve meaning, engagement, and empowerment at work [31].

Second, providers need strategies to mitigate the impact of burnout on their well‐being and patient care. Through the training, we discussed mindfulness strategies that participants could implement immediately upon returning to work. During the in‐depth interviews, we learned that these coping strategies have had a large impact on providers’ experiences of burnout and have created camaraderie as they help each other cope with stress. The survey data 1‐ and 3‐month post‐training revealed relatively low burnout rates again, at 29% and 33%, respectively, showing that the impact of the coping methods on reported levels of burnout endures 3 months after the training. As expressed by one of our interviewees, although the environment has not changed, these numbers show that training participants now have the tools to manage burnout and therefore feel less burdened by it daily.

Finally, institutional approaches alongside individual strategies are necessary to promote provider well‐being [31]. Although individual behavioral coping methods may be helpful in the short term, broader systemic changes are needed to address the core causes of burnout among providers, requiring structural changes and increased funding of the public healthcare system.

### 4.3 Limitations

This study engaged L&D providers across a range of clinical settings (public and faith‐based) in northern Tanzania, making the results generalizable to a range of settings in Tanzania and perhaps beyond. The results, however, must be interpreted in light of the study limitations. One limitation is the length of the quantitative burnout assessment. Although the two‐item measure has been validated previously [23], it remains a more limited evaluation of burnout compared to the full 22‐item Maslach Burnout Inventory. However, the ability to rapidly administer the survey was necessary in our setting. Another limitation is the absence of a comparison condition to conclusively evaluate the impact of the intervention on provider burnout. However, the interviews pointed to an improvement in provider burnout as a result of the training content. Finally, due to the small sample size, we were not able to stratify data by demographics or professional characteristics.

## 5 Conclusion

Burnout is a global issue with harmful impacts on healthcare providers, patient outcomes, and health systems. In low‐resource settings, burnout is exacerbated by workforce shortages, high patient loads, and limited resources. Yet the prevalence and impact of burnout among healthcare providers in Sub‐Saharan Africa have rarely been studied. This study sheds light on the experience of burnout in Tanzania and can help provide guidance on future assessments of burnout and interventions in this setting. Increasing awareness of the issue is crucial to enable providers to recognize burnout, and further investment in individual and system‐wide strategies to reduce burnout is needed.

## Author Contributions

**Virginie Marchand**: conceptualization, writing–original draft, methodology, formal analysis, investigation. **Melissa H. Watt**: conceptualization, writing–review and editing, methodology, formal analysis. **Linda M. Minja**: formal analysis. **Mariam L. Barabara**: conceptualization, investigation. **Olivia R. Hanson**: writing–review and editing. **Janeth Mlay**: investigation. **Maya J. Stephens**: writing–review and editing. **Blandina T. Mmbaga**: writing–review and editing. **Susanna R. Cohen**: conceptualization, writing–review and editing, methodology, formal analysis.

## Ethics Statement

Written informed consent for participation in the MAMA training, completion of surveys, and participation in interviews was obtained from all participants prior to the initiation of the study. The study received IRB approval by the ethical review committees at the University of Utah (Protocol 00143918), Kilimanjaro Christian Medical Center (Protocol 2056), and National Institute for Medical Research in Tanzania (Protocol 3853). The evaluation of the MAMA training is registered at clinicaltrials.gov (NCT05271903).

## Conflicts of Interest

The authors declare no conflicts of interest.

## Supporting information



Supporting Information

Supporting Information

## Data Availability

A limited, de‐identified dataset is available in the repository of the Inter‐University Consortium for Political and Social Research (ICPSR‐194883).
