# Supplementary material for: Burnout Among Labor and Birth Providers in Northern Tanzania: A Mixed‐Methods Study
Source: Public Health Chall. 2024 Dec 5;3(4):e70014. doi: 10.1002/puh2.70014 (PMC12039629; doi:10.1002/puh2.70014)
Supplement: Supplementary file 1 — Supporting Information [file PUH2-3-e70014-s001.docx]

Supplemental table 1. Consort diagram

**Invited to participate**

**(n=60)**

**Completed 3 Month Assessment**

**(n=59)**

**Completed 1 Month Assessment**

**(n=55)**

Changed jobs (n=1)

Maternity leave (n=1)

Busy with clinical duties (n=2)

Loss to follow up (n=1)

**Completed Immediate Post Assessment**

**(n=60)**

**Attended MAMA training**

**(n=60)**

**Completed Baseline Assessment**

**(n=60)**

**Enrolled**

**(n=60)**
