# Supplementary material for: Burnout Among Labor and Birth Providers in Northern Tanzania: A Mixed‐Methods Study
Source: Public Health Chall. 2024 Dec 5;3(4):e70014. doi: 10.1002/puh2.70014 (PMC12039629; doi:10.1002/puh2.70014)
Supplement: Supplementary file 2 — Supporting Information [file PUH2-3-e70014-s002.docx]

**In-Depth Interview Guide for MAMA Training Participants**

Sub-Section of Longer Interview Guide

1. **Burnout**
2. *Prevalence of burnout*

During the MAMA training, we talked about burnout. Burnout results from chronic stress and emotionally intense work, and it is characterized by emotional exhaustion, increased callousness towards patients, and strong feelings of frustration and powerlessness in patient care.

- 1. **Do you feel like burnout is common among your colleagues?**
     - **IF YES: Please tell me what burnout looks like. (Probe for specific examples.)**
     - **IF NO: Why do you think that your colleagues don’t experience burnout?**
  2. **IF YES: Before the training, were feelings of exhaustion or frustration discussed between colleagues?**
     - **IF YES: How were these feelings described?**
     - **IF NO: Why do you think this experience was not discussed, even though the feelings are present?**

1. *Activity:*

We did an activity during the training where we invited everybody to stand along a line according to their level of burnout (show picture).

- 1. **What were you thinking about as you chose your place in the line?**
  2. **How did it feel to participate in this activity?**
  3. **How did it feel to watch your colleagues line up?**
  4. **How did this activity and discussion change your understanding of you and your colleagues’ experience of burnout at work?**

1. *Discussion of burnout at work*
   1. **Is burnout something you’ve talked about with other providers since the training?**
      - **IF YES: How have you talked about it?**
      - **IF NO: Why have you not talked about it?**
2. *Explaining the low reported rates:*

We also asked about burnout on the survey. As you can see from this data, when we asked about burnout *before* the training, not many providers reported burnout. When we asked about burnout immediately after the discussion and activity in the training, more providers reported burnout.

- - - Probe based on their response.
  1. **What do you think explains the low levels of burnout when we first asked these questions to providers?**
  2. **What do you think explains why providers reported *more* burnout after discussing burnout together and doing the activity?**
  3. **As you see here (show graph), when we returned for the in-situ one month later, we asked the same questions about burnout and ratings were low again.**
     - **Why do you think providers continue to rate low levels of these feelings of burnout even though in reality many have high levels of burnout in the work environment?**
  4. **What do you think contributes to burnout in providers?**
  5. **How do these feelings of burnout affect the delivery of care to women during labor and delivery?**
